# Bridging the gap between health and justice

**DOI:** 10.1186/2194-7899-1-4

**Published:** 2013-11-18

**Authors:** Lior Gideon

**Affiliations:** Department of Law, Police Science & CJA, John Jay College of Criminal Justice, New York, USA

What does health have to do with justice? Everything! More than seven million people in the United States are currently under some form of criminal justice supervision, and control (whether in jail, prison, or on probation or parole), and they have somatic and mental health issues in rates that are much higher than those found in the general population. Ignoring these issues will contributes to health disparities and negative outcomes and this will be unjust—not only for those individuals—for the communities in which they live (or will live, upon release), and for human kind in general. As this article will show, the health needs of this population are also of pmount importance to those within specific communities, even if they are not currently involved with the criminal justice system. It is thus critically important to bridge the gap between the fields of public health and criminal justice by promoting an interdisciplinary discourse that brings together professionals and scholars from both ends of the justice and health spectrum; and thus better serve society, and address health disparities issues that affect health and justice.

Criminal justice practitioners and public health workers may, at first glance, seem to be in unrelated (sometimes even opposing) fields, but in fact, they share many of the same challenges, as they both deal with human beings, environments, and public safety. Yet these two fields have long operated in pllel universes, and only recently has that approach begun to change. Scholars, practitioners, and policy makers from both sides have started to share ideas and information in an effort to create a better response to rising challenges that concern both fields. So far, most of the work has been related to incarcerated offenders and their rehabilitation and reintegration, but health-related issues are also relevant for examining and intervening with non-incarcerated criminal justice populations.

As Levy (2007) states, “…public health practices inform and empower individuals and communities, and create healthy environments through the use of evidence-based strategies and accountable mechanisms.” (p. 74) Similar practices should be considered an essential part of criminal justice as well, so that both fields engage in the science of promoting public safety while preventing disease and injury, prolonging and improving life, and promoting health.

To this end, the journal of *Health & Justice* aims to promote discussions and collaborations to advance understanding and present solutions to the challenges found in the rather large space where health and justice intersect. As a starting point, this article aims to demonstrate how public health issues are inherent in numerous aspects of the criminal justice system. Then it will offer a conceptual framework for applying many of the fundamental principles of public health to the realm of criminal justice—whether specifically to those who are incarcerated or otherwise under supervision of the justice system or, more broadly, to a broader range of social ills.

## The nexus of health and justice

Prison health care issues should not remain behind bars. Many incarcerated individuals enter jails and prisons with myriad health problems, often suffering from comorbidity of symptoms and illnesses. The prevalence of some chronic diseases, substance abuse, and mental health issues is much higher among incarcerated offenders compared with non-offenders (Dretsch 2013; Freudenberg 2001; Watson et al. 2004).

Left unaddressed, many such issues may result in detrimental community consequences. For example, individuals infected with tuberculosis (TB) can infect healthy people upon release, as the disease is airborne and easily spread in high-density population areas. Similarly, other sexually transmitted diseases (STDs) that are not detected and treated during incarceration can cause an epidemic later in certain communities to which such individuals are released. Generally speaking, the health issues of those individuals who are involved with the criminal justice system may become impediments to their rehabilitation, reentry, and reintegration. Thus, the health care of incarcerated offenders ought to be at the top of the agenda, not just for the sake of the prisoners themselves, but because untreated problems can easily affect a large number of people, and even entire communities.

Identifying the medical and psychological needs of incarcerated offenders while they are imprisoned is of great importance. For many offenders, their incarceration period provides a good starting point for addressing their unmet needs and risk factors, as well as their risk to the community. By addressing and solving the immediate health problems of incarcerated offenders, a greater good is achieved. When individuals who, upon release, are healthier than when they were sentenced and hold more positive attitudes toward personal health, the wider community benefits (Watson et al. 2004).

In addition, the prevalence of contagious diseases such as HIV and hepatitis is much higher among incarcerated offenders compared with those in the community at large. If this disparity is not addressed, Macalino et al. (2004) reports that:

…concerns exist that jails and prisons could serve as reservoirs that could amplify transmission of infectious diseases in the wider community as inmates who become infected behind bars are released. Such reservoirs would be formed by the high prevalence of infections such as HIV, hepatitis B virus (HBV), and hepatitis C virus (HCV) among inmates, particularly those with a history of injection drug use (p. 1218).

A recent report by the federal Office of Justice Programs reveals that at the end of 2010, state and federal prisons held 20,093 inmates who had HIV or AIDS, which reflects a rate of 146 per 10,000 inmates (Maruschak 2012). These rates are significantly higher compared with the rates in the general population and can be attributed to injection drug use, unprotected sexual activity, and other risky behavior that is prevalent among incarcerated offenders. In addition to these bleak statistics, another unknown number of people are contracting the disease while incarcerated because many inmates are not diagnosed upon admission, and thus the threat to communities is increasing even more (Koulierakis et al. 2000; Macalino et al. 2004).

Further, the nexus of mental illness, delinquency, and criminal behavior is much documented. Following the deinstitutionalization movement of the 1950s and 1960s, many facilities for the mentally ill reduced the number of beds available, shifting the responsibility for the care of those with mental illnesses, some of which pose a danger to society, to the criminal justice system, in particular to corrections (Gideon 2013). Such an increase in mentally ill individuals behind bars demands a high level of professionalism within jails and prisons, and also among members of law enforcement and those responsible for sentencing. Some even argue that modern prisons have become the “new asylum” (Arngo 2002; Fagan and Ax 2011). It is likely no coincidence that Noonan (2010) reports that the number of deaths in local jails has increased each year. Examining national mortality data from jails across the United States between 2000 and 2007, Noonan found that death from illness, including AIDS, accounted for more than half (53%) of all deaths in local jails. Noonan further found that heart disease was another leading cause among deaths in local jails (42%), with suicides the leading cause of unnatural death. This last cause of death strongly relates to mental illness. Further, the report shows that more than half of all jail deaths occur within the first month following admission.

## Sentencing and health concerns

Many of the chronic illnesses among adults in the general population are shared by those behind bars. Young et al. (2009), state that criminal offenders are among the unhealthiest people on most measures of physical and mental illnesses. Their life expectancy is low, and they are more vulnerable to all chronic and acute health conditions (Binswanger et al. 2007). Thus, they have a desperate need for quality care. Freudenberg (2001) found that individuals entering jails and prisons are more likely to suffer from a chronic medical condition, substance abuse, or mental illness than members of the general population. Watson and his colleagues (2004) argue that the nature of most health problems experienced by prisoners indicates a link between the health problems that “…prisoners bring with them to prison and those from which they are at risk” (p. 122). This is mainly due to the fact that health issues are lower on the priority list for many of those incarcerated. Specifically, previous risky behavior and long histories of dire hygiene, combined with a disadvantaged environment and later prison environment, all correlate with health deficiencies. Dretsch (2013) argues that about 40% of all federal inmates, local jail inmates, and state inmates have at least one documented chronic illness (also see Wilper et al. 2009). Further, a study conducted in the United Kingdom found that male prisoners tend to consult doctors about three times more often than a demographically equivalent community population, and consult health care workers 77 times more, compared with men in the larger community; this is also true for incarcerated female offenders, who consult with prison doctors about three times more than females in the community do, and with health-care workers about 59 times more frequently (Marshall et al. 2001).

Consequently, an important part of the presentencing report and consequent sentencing should include a detailed evaluation of the defendant’s physical and mental health. Such information will enable a more effective sentencing while maintaining the best interest of the community and the individual. In that regard, it is also vital to acknowledge that preexisting health conditions are often linked to other physical and mental conditions. One example is substance abuse and mental health problems; likewise, intravenous methods of self-administration of substances may increase the risk of contracting HIV or hepatitis B or C.

On another level, sentencing should take into consideration the potential harm of the actual punishment and its consequences not just for the defendant, but also for those others affected by the sentence, including the defendant’s family and the community at large. For instance, sentencing a young, nonviolent offender to incarceration could do more harm than good, with detrimental effects on the individual, his close family, and the community. This effect is compounded if he is suffering from substance abuse and/or mental health problems.

## Intake and screening

A formal assessment of risk should identify those events and exposures that may be harmful to individuals and social environments—both the prison or jail and the community at large. Specifically, the intake process should identify those risk factors that may later have a devastating impact on public health and community safety. Community agencies and public health professionals can use historical data and trends of the effect of mass incarceration, for example, to estimate the risk offenders may pose to their home community upon release, taking into consideration the high rates of substance abuse, mental illness, STDs, and HIV/AIDS in a given population. Yet systematic management of risk should not be limited to individual offenders; instead, it should be expanded to include the examination of facilities and communities. Overcrowded prisons are a breeding ground for diseases transmitted through air, food, and water (Schneider 2011).

Assessing risk and needs at intake is also important if we want to reduce the potential harm to communities and general society. According to Levy (2007), risky behaviors “are, and will continue to be, a part of society” (p. 75) and thus must be identified at early stages, so as to prevent greater harm. For example, young adults who enter jails are at a much higher risk for contracting STDs, as they often engage in risky behavior and unprotected sex with multiple partners and tend to use illegal substances (Centers for Disease Control and Prevention [CDC], 2011); however, few jails offer any screening for common STDs such as *Chlamydia trachomatis* and *Neisseria gonorrhoeae* infections. Untreated, these diseases can lead to much greater health conditions, including increased risk of contracting HIV infection (Wasserheit 1992).

Health screening at admission to a correctional facility and as a routine part of correctional primary care both protects the facility’s population and staff and delivers appropriate prevention to otherwise underserved individuals (Lee et al. 2007). In addition, health screening at admission has the ability to further contribute to public safety, because individual inmates are assessed for illnesses and their risk of infecting others upon release. Consequently, it is of great importance to use the period of incarceration “to impact public health using evidence-based screening of high-risk individuals who do not otherwise have access to routine preventive care” (Lee et al. 2007, p. 249). Lee and his colleagues further argue that “effective screening in jails and prisons is generally a cost-effective approach to improving population health.”

Thus, the aim of intake assessment at early stages of the criminal justice process, and in particular before sentencing and incarceration, is to identify those risk factors or diseases that can be treated by providing targeted interventions. This is in addition to identifying mental health problems as well as static and dynamic factors that may affect future criminality. Accurate detection of disease during the early stages of admission can meaningfully change the course of disease and reduce the risk of infection and transmission to criminal justice practitioners and the community, and thus will make early screening meaningful (Lee et al. 2007). Early screening and assessment of needs and risks is also of great importance for the reduction of mortality incidents associated with criminal justice agencies and actions. Adequate screening for depression, substance and alcohol abuse and withdrawal from them, and suicidal tendencies will help minimize these leading causes of mortality among incarcerated individuals (Lee et al. 2007). From this point, assessing risks and devising ways to properly target them can improve decision making and policy (Pickett and Hanlon 1990).

## Rehabilitation, reentry, and reintegration: health impediments

Reentry and reintegration can be stressful to those released from incarceration. The challenges of obtain housing, secure employment and gain access to health care are essential to successful reintegration. According to Levy (2007), reintegration after release from incarceration poses the ultimate challenge to an individual’s health care: “Ex-prisoners with previous histories of mental illness or drug dependence present particular risks of early death—either from drug overdose, suicide, or violence” (p. 83; also see Binswanger et al. 2007; Drucker 2011). Although incarcerated inmates do receive medical attention during their period of incarceration—some will argue that incarcerated individuals receive better medical treatment than the one available to them in their communities—not all health conditions can be addressed during incarceration, and even when addressed, the follow-up after release is unknown. Leaving health conditions untreated has a harmful effect on individuals’ ability to positively and meaningfully engage and complete rehabilitation programs that prepare them for reentry and reintegration. Health conditions such as STDs, as discussed earlier, may have devastating consequences when they spread to correctional staff and members of outside communities. Many such diseases are highly infectious, but adequate treatment can reduce the risk of infection, control the spread, and at times even provide a cure. This is the case for infections such as chlamydia and gonorrhea. More deadly infectious diseases such as TB and HCV can also be controlled, in the process improving an individual’s life while reducing greater harm. When such diseases are controlled, individuals have better chances of complying with substance abuse intervention, educational training, and other attempts at rehabilitation and preption for reentry. Correctly diagnosed health conditions are crucial for the transition from incarceration to the community, and should be included in the paperwork and discharge planning that takes place before the inmate is released. This step is critical to reintegration, as many released offenders do not follow recommended treatment for a host of reasons, most of which relate to the social and cultural environment into which they are released. As such, untreated health issues impede the released offender from finding and obtaining employment and consequently from becoming fully engaged in the community. Moreover, untreated infectious diseases can spread to other members of the community, creating an epidemic.

## Discharge planning and continuum of care

With high rates of highly infectious diseases such as HIV/AIDS, TB, HCV, and other sexually transmitted diseases, as well as mental illness, prison populations can pose a threat to public health when offenders are released without adequate planning. Indeed, discharge planning should be a top priority in the battle to maintain public health and safety.

Discharge plans provide the critical link between prison-based intervention and the transition from incarceration to the community. As noted by Gideon and Sung (2011), the primary goal of a discharge plan is to link the incarcerated individuals with appropriate health and human service providers in the community. In that regard, discharge planning is an essential effort that directs soon-to-be-released inmates in the right direction so they will be able to receive the help and care they need, before they recidivate. In that regard, Gideon and Sung state that “…the entire rehabilitation, reentry, and reintegration process should be coordinated with the community to promote collaborative efforts that will ensure a continuum of care during the reentry process for the purpose of successful reintegration” (p. 404), and in order to contain potential health risks and promote safety.

In fact, the health field provides a good model for discharge planning, where it is common practice—for instance, when someone is released from hospitalization. In this context, a discharge plan—which health practitioners refer to as secondary or tertiary prevention, after the primary prevention provided by direct medical treatment—maintains the goals of promoting health and maximizing quality of life for those harmed by a disease. According to Drucker (2011), in the context of incarceration, this practice of discharge planning constitutes the “tertiary prevention” of potential epidemics by simply minimizing the suffering and disability of the formerly incarcerated. It also has the ability to reduce mortality among this vulnerable criminal justice population while improving quality of life. In the following section of this article, the importance of this stage is further examined by applying the “chain of infection” model to better understand how a public health approach to criminal justice can greatly advance the field.

## A public health approach to criminal justice

Public health focuses on prevention, the occurrence of diseases, and the general promotion of health and safety. In that regard, public health takes a more proactive approach compared with medicine, and also compared with the traditional criminal justice system, which tends to be highly reactive. Through the basic scientific method of epidemiology—which Merrill (2013) defines as “…the study of distribution and determination of health-related states or events in human population and the application of this study to the prevention and control of health problems” (p. 2)—public health workers can identify and control the risk of infection of a potential plague. A similar approach can be applied to criminal justice research and policy: researchers are called to investigate and identify the causes of “social ills” that affect public safety and health. By looking at criminal justice through the lens of public health, researchers can better evaluate the effectiveness of current practices and how they reduce criminality, mass incarceration, and the devastating effects that follow them.

Through the use of systematic observation data and other vital statistics, researchers can not only identify the cause of a social problem but also devise an evidence-based practice to successfully target that problem, as was done in London during the nineteenth century to identify and target the cholera epidemic (Snow 1855). Yet criminal justice scholars and criminologists are not concerned with epidemics caused by a specific pathogen, such as the one Dr. Snow identified as cholera. Instead, they are concerned with both literal illnesses and more metaphorical ills—that is, other types of epidemics, ones that may have an equally devastating effect on entire communities and societies, as illustrated by Drucker (2011). To borrow health terminology, many offenders in the criminal justice system suffer from real pathogens (TB, HCV, etc.) as well as what could be called social pathogens (poverty, social decay, etc.)—a complex variety of comorbidity.

Using a public health approach, the first stage of properly addressing health issues within the criminal justice population is to define the prevalence of health problems that such individuals experience. Next, it is important to identify the risk factors associated with such health issues. The following stage should be to acknowledge that those individuals will return to their communities after release, and from there examine and develop an appropriate community-based intervention that will minimize the severity of their illnesses, while reducing the odds of reoccurrence and magnitude of the symptoms (secondary prevention), and at the same time prevent these health issues from spreading to the larger community. The process cannot be completed without properly monitoring such interventions, where assessment and evaluation are needed not just to examine the outcomes, but for further identification of elements that may have been overlooked in the process. It is at the point of reentry and reintegration that tertiary prevention is needed to improve the quality of life of those who went through the criminal justice system, while minimizing the potential damage to both individuals and communities (as discussed by Drucker 2011; also see Mauer and Chesney-Lind 2002). In order to illustrate the importance and relevance of such an approach, the following pgraphs demonstrate the prevalence of some health-related issues and concerns among the incarcerated population.

### Prevalence of traumatic brain injury

Many incarcerated individuals are living with Traumatic Brain injury related problems that complicate their management and treatment while in jail or prison. A report by the CDC (2007b) indicates that the prevalence of traumatic brain injuries (TBI) among criminal justice population is significantly higher than that found in the general population. Studies conducted between 1998 and 2006 found that the prevalence of traumatic brain injuries in the jail and prison population ranges between 25% and 87% (Morrell et al. 1998; Schofield et al. 2006; Slaughter et al. 2003). In a more recent study conducted in the U.K., Williams et al. (2010) found that adults with TBI were on average younger at entry into custodial systems compared with those who did not suffer such injuries, and the same population reported higher rates of recidivism, as measured by repeated offending. Additionally, Williams and his colleagues also found that those who suffered from TBI reported longer incarceration time in the previous five years. Aside from the clear association with criminal behavior, TBI is known to affect cognitive behavior, impede thinking and speech, and, in the most severe cases, permanent disability, which may later affect chances of finding employment and thus successful reintegration. This clearly explains the high rates of recidivism documented by Williams and his colleagues.

### Prevalence of blood-borne and sexually transmitted diseases

Blood-borne and sexually transmitted infectious diseases (syphilis, chlamydia, gonorrhea, hepatitis, and HIV/AIDS) are known to be much more prevalent among criminal justice clients than in the general population (Macalino et al. 2004; Maruschak 2012). In a recent report published by the CDC (2011), two highly infectious sexually transmitted diseases were examined for prevalence and detection in 12 large jails in the United States. The report shows that adults entering jails have much higher prevalence of both chlamydia and gonorrhea; in particular, chlamydia infection among female jail inmates has been higher than that observed in the general population. Yet many correctional institutions do not perform screening for STDs, and when screening is performed, it is not clear how many inmates actually receive treatment (Macalino et al. 2004; Maruschak 2012; CDC 2007b). The potential dangerous effect of such ignorance and neglect may result in a situation in which, according to the CDC report, in “…both men and women, untreated infection can promote both the acquisition and transmission of HIV” (p. 3).

As noted earlier in this article, a recent report by the Bureau of Justice Statistics, state and federal prisons held 20,093 inmates diagnosed with HIV or AIDS. Although the rate of HIV/AIDS, one of the more deadly infectious diseases in recent years, has been declining among federal and state prison populations (Maruschak 2012), public health and community safety concerns are still valid, as many of those infected will be released back to their communities. In fact, the BJS report numbers are not accurate, as HIV/AIDS testing on entering inmates is not mandatory, and screening is not regularly performed (Drucker 2011). As a result, there are uncounted numbers who are infected with the virus but are most likely not aware of it, and thus pose even greater risk to their families and home communities upon return.

As for the hepatitis C virus (HCV), its prevalence among the incarcerated is between 10 to 25 times higher than in the general population. According to Dretsch (2013), it is estimated that anywhere between 15% and 40% of inmates incarcerated in U.S. jails and prisons are infected with the virus, “…despite the fact that the rate of infection among the general population is only 1.6%” (p. 121). Consequently, mortality rates tend to be higher in the criminal justice population as well.

### Mortality rates

According to a report by the Bureau of Justice Statistics documented a 22% increase in the number of deaths in local jails between 2000 and 2007 (Noonan 2010). The leading cause of death is suicide (29%), followed by heart disease (22%), intoxication (7%), and HIV/AIDS, which accounts for 5% of deaths in local jails. Noonan stated that more than 50 different medical conditions were reported by local jail administrators as the causes of death among jail inmates. In a later study, Noonan (2012) documented a decrease in the number of deaths in both jails and state facilities, with 4,150 documented deaths in 2010. In that year, the six leading causes of inmates’ death were cancer, heart disease (increased in rate since 2009), liver disease, respiratory disease, HIV/AIDS, and suicide, which remained about the same through the years—about 30% (Noonan 2012). Mortality rate are was also found to be higher among former inmates. Specifically, Binswanger and her colleagues (2007), who examined and compared mortality rates among released inmates from the Washington State Department of Corrections, found that the risk of death among those released from incarceration is 3.5 times higher than that of non-previously incarcerated residents of the same age, gender and race.

Mortality rates are also higher among untreated substance abusers that are released from incarceration without any meaningful treatment. For example, Binswanger and her colleagues (2007) examining a cohort of inmates released found that during the first two weeks after release the risk of death among former inmates was 12.7 times higher compared with other state residents, “…with a markedly elevated relative risk of death from drug overdose…” (p.157). In a later study, Drucker (2011) confirms such result arguing that death often comes as a result of overdose, usually within the first few weeks after release (Drucker 2011). Precise rates of drug overdoses are hard to come by, as they are not specifically tracked by the CDC, but Paulozzi et al. (2006) propose that CDC reports on poisonings can reflect the truth, as often, such incidents are the result of nonmedical use of various substances, some of which are illegal, consumed by individuals with histories of drug abuse. According to a 2007 CDC report, the death rate by poison in the general population rose by almost 80% between 1999 and 2005, with some states reporting higher increases (CDC 2007a).

## Health concerns beyond incarceration

So far, this article has discussed, for the most part, how public health issues overlap with incarcerated offenders. But this is not the only part of the criminal justice system with special health concerns. Consider, for instance, how the last couple of decades have introduced us to new types of crimes, terrorism, and natural disasters (the most recent being Hurricane Sandy in the New York tri-state area in fall 2012). Bioterrorism is now a well-documented phenomenon. Incidents such as the anthrax attack of 2001, the Aum Shinrikyo cult sarin gas attack, and the salmonellosis outbreak in 1984 in Oregon are just few examples out of many more where health and justice collaborations are called into action. Law enforcement and other arms of the criminal justice system often are the first to respond to such incidents, and it is important that they are prepared to deal with such situations where public health is at risk. The presence of hazardous materials should be taken into consideration. An unfortunate proof of this came from the collapse of the World Trade Center towers in New York City during the 9/11 terror attack; many first responders were exposed to toxins and asbestos, which later caused lung cancer and other terminal illnesses. In a cohort study that followed 3,700 first responders working at the site, now known as Ground Zero, Webber et al. (2009) found that three years after the initial exposure, about 40% continue to suffer some respiratory symptoms (e.g., dyspnea, wheezing, rhinosinusitis), as well as gastroesophageal reflux disease (GERD). In similar scenarios, it’s important to remember that exposure to toxins or pathogens can affect not only those directly affected by the attack or natural disaster, but also many others who simply approach the scene to offer help and find family members. This is a concern not just for the health professionals but also for those first responders and criminal justice representatives who must isolate the area and minimize the damage. These are issues that need to be explored further.

## The epidemiological model

This paper has shown how public health concerns should not be ignored inside prisons and jails, or among other criminal justice professionals and first responders, and it has laid out a few specific suggestions for applying the ideals of public health in a criminal justice setting. But for health and justice practitioners to work together and conduct research in the long run, it is also necessary to have a broad theoretical framework with which to approach major issues in the field.

**Figure 1:**
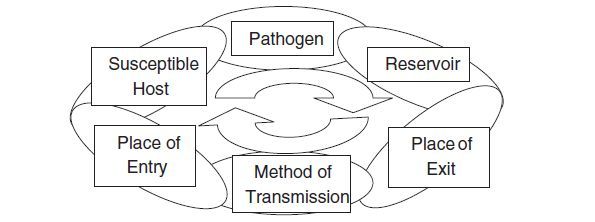
**The cycle of infection.**

One of the most basic and important scientific tools of public health is the epidemiology, and the epidemiological model (Figure 1), which is used to identify infectious diseases and control their spread. Applying the vocabulary and concepts of epidemics to the criminal justice sphere, will create a more leveled ground upon which health and justice scholars and practitioners can better collaborate to devise new studies, propose solutions and advance policies.

The first stage in preventing an epidemic is to identify the *pathogen*, which may take various forms. In the context of the criminal justice system, the pathogen is not always an STD or a virus such as HCV or HIV/AIDS. If we consider health problems more metaphorically, the pathogen can also be substance abuse or mental health issues, even though these are not infectious in the most literal sense.

Identifying the pathogen should be done during the intake and screening stage, when an offender enters the criminal justice system. This is an important part of any criminal justice function, and in particular when dealing with the offender population. As established earlier in this article, this population is characterized by significantly worse health than the non-offender population, with higher rates of infectious diseases, some of which are terminal. Controlling such individuals and their health issues should be the system’s highest priority, with assessment at the point they enter and throughout their involvement in the system, until their release back in to the larger population.

The next stage is to look at the *reservoir*, the place where the pathogen lives and multiplies. In the criminal justice system, jails and prisons are the most obvious reservoir: In their confines, many air-, food-, and blood-borne pathogens can cause fatal infectious diseases such as HCV, HIV/AIDS, STD and TB. It is thus critical to prevent people from unnecessarily being placed into this reservoir. For instance, whenever possible, young, first-time offenders should be diverted from the system with the aim of preventing them from unnecessary exposure to the harmful pathogens found in prisons. Criminal justice agencies must collaborate with health professionals and various agencies in the community to identify those individuals who can benefit from non-incarcerative sentences, and thus prevent them from being placed in the reservoir (that is, prison) where they may contract the pathogen.

After this, one looks at the *method of transmission*—that is how our identified pathogen travels from one reservoir to another or, differently put, from one community to another. Stopping the spread of a pathogen at this stage requires intervention with practical advice, services, and monitoring. In the context of incarcerated offenders, as proposed earlier in this article, it is essential to devise an appropriate discharge plan for each individual and then create transition programs from the criminal justice setting to the community, where follow-up and adequate intervention and treatment are available.

As for the *susceptible host*, where the pathogen may land if it is not adequately controlled, that is easily identified using the descriptive observations found in the science of public health (i.e. epidemiology). The incarcerated population is mainly young, less educated men from poor, deprived minority communities. It is no surprise that upon their release, former prisoners return to the same communities (see Petersilia 2001; Travis 2005), and by doing so, they cause collateral damage to their communities of origin (Clear 2007). Such a pattern, as documented by the Urban Institute (La Vigne et al. 2003), causes what Gideon (2010) calls a “desertification process,” which makes those communities more susceptible to health issues that were untreated during incarceration. One need only take into consideration the high rates of teenage and unmarried pregnancies in such communities, the lack of quality medical care, and the often poor environmental hygiene (Schneider 2011) to better understand how infected individuals returning home pose a great risk upon reintegration. When pathogens (health issues) in jails and prisons go untreated, the chain of epidemic is unbroken, and these host communities then become a larger reservoir, in which the identified pathogens can often spread just as well as within the walls of prisons. And if the cycle continues unbroken too long, the pathogens can spread beyond prisons and disadvantaged communities and into larger portions of the population, possibly producing a major epidemic.

From the above, it is crucial that criminal justice professionals, in collaboration with health practitioners and community organizations, address the challenges faced by the justice-involved population—that is, those most likely to be incarcerated, according to demographic risk—and devise strategies to reduce that risk and minimize the damage for both individuals and communities. By targeting and disabling these larger habitats where potential harmful pathogens breed (which is to say, by alleviating poverty and increasing education, as well as treating specific health problems), the risk to public health and safety can be decreased.

To apply this model of the chain of infection, and to break the cycle while promoting public health and safety, studies should aim to answer the basic questions of who, where, when, and how by examining patterns of infection, recidivism, and criminal justice–related mortality. It is also important to take environmental factors—both physical and social, such as poverty, exposure to toxins, condensed and decayed urban areas, high rates of out of wedlock births, and the like—into consideration. The cycle witnessed by the criminal justice population is complex. But by following this fairly straightforward pattern, we will at least be able to minimize and prevent potential collateral damage to communities and future generations.

## Conclusion

We need a thorough research agenda that will enable us to examine best practices of diagnosis, treatment, and prevention of those ailments that relate to crime and health. The time has come for more resources to be allocated for such goals to be achieved. By doing so, not only will we be dealing with our offenders in a more humane manner, but our communities will enjoy healthier and safer environments, while we do justice to those who are most vulnerable in our society.

The sword and scale of Lady Justice should not be just for sentencing. Justice will be served when the best interests of both the offender and the community are served, when balance is restored and health and justice become a higher moral calling, one that we must pursue and maintain. Applying public health and epidemiological knowledge to the research and understanding of many criminal justice issues is a valid and necessary approach. By the same token, public health can benefit greatly from examining criminal justice policies and practices and the manner in which they affect health and communities.

The balance between justice and community safety is intimately connected with the health of those incarcerated, and thus it becomes an essential issue in considering the impact of incarceration on larger public health, of both individuals and communities (Levy 2007). According to Beauchamp (1976), “public health should be a way of doing justice, a way of asserting the value and priority of all human life” (p. 8), regardless of what one did to merit incarceration. Health care for criminal justice population should be considered a fundamental right, an essential component of social justice (Schneider 2011). According to Pickett (1989), “…public health, unlike virtually all other important social efforts, is dependent on its ability to obtain the participation of other agencies to solve its problems” (p. 399). It is with these insights that *Health & Justice* strives to bring together the different sectors that deal with the well-being of individuals and communities to promote health, safety, and justice through research and discussion. Successful health and justice policies should involve all those who are interested in bettering our communities.

Health and justice are not that different from each other; they are two sides of the same coin, sharing a mutual aim of providing public safety. It is thus imperative that the two disciplines collaborate and learn from each other, not just in matters that pertain to the incarcerated population, but at all levels, with one aim in mind: promoting the health and safety of our communities. This is justice, and we should do everything in our power to restore and maintain it.

## Authors’ original submitted files for images

Below are the links to the authors’ original submitted files for images. Authors’ original file for figure 1
