# Supplementary figures and images for: Bridging the gap between health and justice
Source: Health Justice. 2013 Nov 18;1:4. doi: 10.1186/2194-7899-1-4 (PMC5120658; doi:10.1186/2194-7899-1-4)

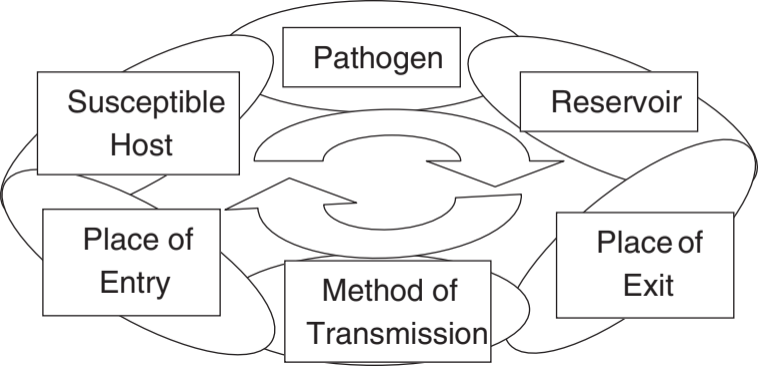

Supplement: Supplementary file 1 — Authors’ original file for figure 1 [file 40352_2013_1_MOESM1_ESM.pdf]
